# The application of different machine learning models based on PET/CT images and EGFR in predicting brain metastasis of adenocarcinoma of the lung

**DOI:** 10.1186/s12885-024-12158-0

**Published:** 2024-04-11

**Authors:** Chao Kong, Xiaoyan Yin, Jingmin Zou, Changsheng Ma, Kai Liu

**Affiliations:** 1 Department of Graduate, Shandong First Medical University, Shandong Academy of Medical Sciences, Jinan, China; 2 Department of Radiation Physics, Shandong Cancer Hospital and Institute, Shandong First Medical University, Shandong Academy of Medical Sciences, 250117 Jinan, Shandong Province China; 3 Department of Head and Neck Comprehensive Radiotherapy, Affiliated Tumor Hospital of Xinjiang Medical University, 830000 Urumqi, China

**Keywords:** PET/CT radiomics, Machine learning model, Lung adenocarcinoma, Brain metastases

## Abstract

**Objective:**

To explore the value of six machine learning models based on PET/CT radiomics combined with EGFR in predicting brain metastases of lung adenocarcinoma.

**Methods:**

Retrospectively collected 204 patients with lung adenocarcinoma who underwent PET/CT examination and EGFR gene detection before treatment from Cancer Hospital Affiliated to Shandong First Medical University in 2020. Using univariate analysis and multivariate logistic regression analysis to find the independent risk factors for brain metastasis. Based on PET/CT imaging combined with EGFR and PET metabolic indexes, established six machine learning models to predict brain metastases of lung adenocarcinoma. Finally, using ten-fold cross-validation to evaluate the predictive effectiveness.

**Results:**

In univariate analysis, patients with N2-3, EGFR mutation-positive, LYM%≤20, and elevated tumor markers(*P*<0.05) were more likely to develop brain metastases. In multivariate Logistic regression analysis, PET metabolic indices revealed that SUVmax, SUVpeak, Volume, and TLG were risk factors for lung adenocarcinoma brain metastasis(*P*<0.05). The SVM model was the most efficient predictor of brain metastasis with an AUC of 0.82 (PET/CT group),0.70 (CT group),0.76 (PET group).

**Conclusions:**

Radiomics combined with EGFR machine learning model as a new method have higher accuracy than EGFR mutation alone. SVM model is the most effective method for predicting brain metastases of lung adenocarcinoma, and the prediction efficiency of PET/CT group is better than PET group and CT group.

## Introduction

Lung cancer has an incidence of about 11.4% and a mortality rate of about 18% in worldwide [1]. Among them, non-small cell lung cancer (NSCLC) is the most common (80–85%) type of lung cancer histology [2]. Brain is the most common site of distant metastases in advanced lung cancer, with approximately 10 to 30% of NSCLC patients having brain metastases at presentation [3, 4], and approximately 30 to 50% of NSCLC patients still developing brain metastases during treatment [5], of which lung adenocarcinoma is the most common pathological type of NSCLC brain metastasis (about 40%). Due to the lack of effective treatment, brain metastases are the most common and serious complication in patients with lung adenocarcinoma [6], and the leading cause of death in patients with lung adenocarcinoma. Although in recent years, multidisciplinary comprehensive treatment, such as surgery, radiotherapy and chemotherapy, and targeted therapy, has improved the quality of life and prolonged the median survival time of patients with lung adenocarcinoma brain metastases, but the overall efficacy has not met expectations [6].

At present, the diagnosis of brain metastases of lung cancer is based on a combination of clinical symptoms and imaging, but some occult micro-metastases cannot be detected and identified in time, and brain metastases can be diagnosed only when typical clinical symptoms and/or imaging density or signal changes occur, with a certain lag [7]. Therefore, if brain metastases can be predicted based on the high-risk factors in lung adenocarcinoma patients before the appearance of typical brain metastasis imaging signs, more treatment opportunities will be obtained for the clinic. Prophylactic cranial irradiation (PCI) has been shown to reduce the incidence of brain metastases in patients with small cell lung cancer and is the most effective way to slow brain metastases in patients with NSCLC [8–9]. However, PCI treatment for patients with lung adenocarcinoma is not selective, but it increases the probability of complications of radiotherapy, aggravates and reduces the quality of life of patients. The results of a study based on a randomized controlled trial of patients with NSCLC with high-risk brain metastases in stage III.A-N2 who were completely resected showed that disease-free survival (DFS) was 28.5 months and 21.2 months, respectively, in the experimental group (prophylactic brain irradiation) and observation groups (OR = 0.67; 95%CI,0.46–0.98; *P* = 0.037), PCI also showed that PCI could also reduce the risk of brain metastases, with 5-year brain metastasis rates of 20.3% and 49.9% in the experimental group and observation group, respectively [10]. This study clarifies that PCI can be used to prolong DFS in patients with high-risk NSCLC brain metastasis, confirms the significance and necessity of screening patients with high-risk NSCLC brain metastasis for PCI, and brings hope for future PCI treatment research.

Targeted gene therapy has become the main treatment method for patients with advanced lung adenocarcinoma because of its advantages of precision therapy and low side effects. Epidermal growth factor receptor (EGFR) is the most common mutant gene in lung adenocarcinoma, especially EGFR-positive mutations play an important role in the occurrence of brain metastasis in lung adenocarcinoma, but there is still uncertainty and controversy in related studies. Studies have found that systemic inflammatory markers such as lymphocyte percentage (LYM%) and neutrophil to lymphocyte ratio (NLR) have certain value in predicting breast, gastrointestinal, and gynecological tumor metastasis [11–14], but there are few studies on whether they can be used as risk factors for brain metastasis in lung adenocarcinoma. In recent years, in order to clarify the risk factors for brain metastasis in patients with lung adenocarcinoma, many researchers [15–18] have worked on clinical risk factors and information obtained by chest CT imaging to predict brain metastases of NSCLC. However, it is difficult to determine the possibility of brain metastases based on chest CT visual observation and evaluation of clinical data alone. 18 F-FDG PET/CT can provide both anatomical and metabolic information of lesions [19], and can extract radiomics features of PET and CT from the region of interest (ROI), respectively, which makes up for this shortcoming. In order to improve the screening rate of high-risk groups of lung adenocarcinoma brain metastasis, personalized treatment plans were formulated early, so as to improve the prognosis and survival of lung adenocarcinoma patients.

Therefore, this study mainly screened the independent risk factors for brain metastasis in NSCLC patients from the clinical data, tumor markers, EGFR mutation status, PET/CT imaging omics and PET quantitative indexes of lung adenocarcinoma patients, and evaluated the efficacy of six machine learning prediction models for lung adenocarcinoma brain metastasis based on 18 F-FDG PET/CT radiomics combined with EGFR.

## Materials and methods

### Patients and inclusion criteria

We included 204 patients with stage III.-IV. lung adenocarcinoma who visited Retrospectively collected 204 patients (76 with brain metastases) with lung adenocarcinoma who underwent PET/CT examination and EGFR gene detection before treatment in Cancer Hospital Affiliated to Shandong First Medical University from January 2020 to December 2020 and underwent PET/CT examination and EGFR gene testing before treatment in Cancer Hospital Affiliated to Shandong First Medical University, Among them, 76 cases were in the brain metastases group and 128 cases in the anencephaly metastases group.

Inclusion Criteria: 1). According to the eighth edition of the American Joint Committee on Cancer (AJCC), the case was diagnosed with stage III.-IV lung adenocarcinoma; 2). Tumor staging and assessment with or without brain metastases; 3). Patients receive routine examination indicators, PET/CT examination and EGFR gene test before treatment; 4). Not receiving anti-tumor therapy before enrollment examination.

Exclusion criteria: 1). Patients with a history of other malignant tumors and non-stage III.-IV. adenocarcinoma; 2). Inability to stage and assess with or without brain metastases; 3). Those who have received anti-tumor treatment and/or incomplete clinical data; 4). No PET/CT and/or EGFR gene test results; 5). Pure ground-glass nodules without FDG metabolism.

The diagnosis of brain metastases in all patients was made by senior radiologists based on corresponding imaging data and clinical symptoms. All patients except those who died or were lost to follow-up were followed for two years to determine whether they had brain metastases. All of the above information was obtained with the informed consent of the patient.

### Image acquisition

The clinical data of patients with stage III.-IV. lung adenocarcinoma were collected, including gender, age, tumor location, tumor stage, whether there was craniocerebral and extracranial metastasis, and the hematological indicators before the first treatment included WBC, NEU, LYM, LYM%, NRL, CEA, NSE, Cyfar21-1 and tumor EGFR gene status.

PET/CT was acquired on the Sygno, Via system (SIEMENS medical systems). Patients should fast for at least 6 h before the examination. Blood glucose concentrations were measured prior to intravenous 5.5 MBq/kg 18 F-FDG to confirm blood glucose values below 6.6 mmol/L. Image acquisition is performed after urination after lying flat and resting for 1 h after 18 F-FDG injection. CT uses the following parameters: 120 kV, 80 mA, 4.25 mm collimation for correction. Then with 3 min per bed, a PET scan from the head to the thighs is performed immediately. Typically, 6–8 beds are checked depending on the height of the patient. The ordered set expectation maximization algorithm was used to reconstruct the PET data. CT images were used to perform attenuation correction and anatomical localization of PET data.

### Tumor segmentation

**Fig. 1:**
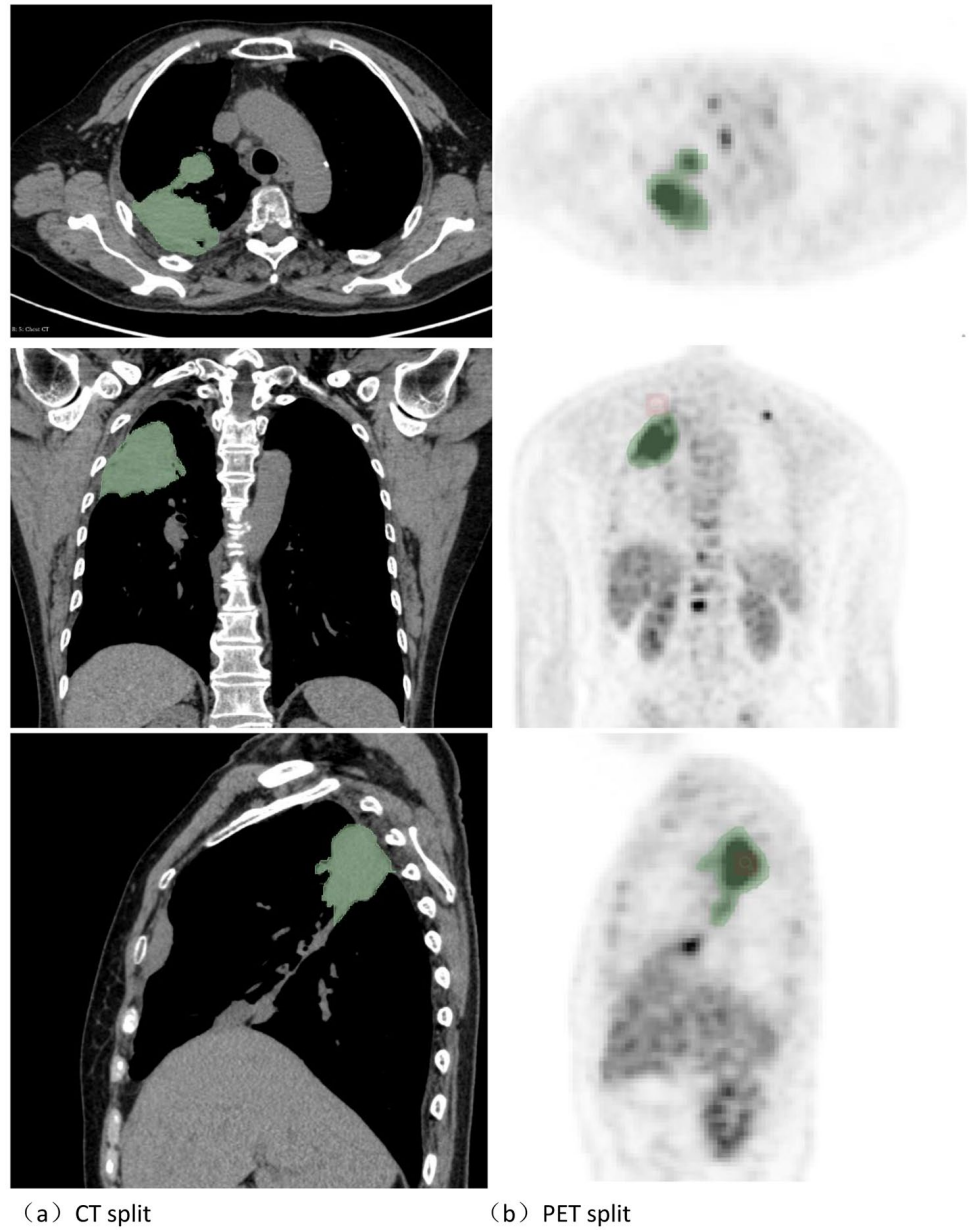
Irregular abnormal density foci in the upper lobe of the right lung. Fig(**a**): Plain CT scan of adenocarcinoma of the upper lobe of the right lung to sketch the image;Fig(**b**) PET sketching image of adenocarcinoma in the upper lobe of the right lung

The regions of interest of CT and PET images were manually segmented and delineated by two experienced radiologists in 3D-Slicer (https://www.slicer.org), preprocessed the images first, resampled the voxel size to 1 × 1 × 1mm^3^, and the CT and PET images were separated by 25 and 0.4 sets of scattered grayscale values, respectively, and normalized the gray values before feature extraction. Develop uniform standards before image delineation and segmentation, and when disagreements are encountered, they are guided by senior imaging experts. In this study, the volumetric segmentation method was used to obtain three-dimensional stereoscopic images of lung adenocarcinoma lesions, which could more comprehensively and accurately show the internal heterogeneity of tumors, which was more conducive to the prediction of brain metastasis of lung adenocarcinoma. At the same time, PET tumor metabolic indexes were obtained, including five tumor metabolic characteristics of SUVmean, SUVmax, SUVpeak, Volume, and TLG (Fig. 1).

### Feature extraction and dimension reduction

The Radiomics plug-in was used to extract representative radiomics features. The CT and PET images extracted 7 types and 851 radiomics features, mainly including shape features (2D, 3D), gray level dependence matrix (GLDM), gray level cooccurrence matrix (GLCM), first order, gray level travel matrix (gray level) based on raw data and wavelets run length matrix (GLRLM), gray level size zone matrix (GLSZM), neighboring gray tone difference matrix (NGTDM). Because the extracted radiomics feature values differ from each other, they are multidimensional data. Therefore, standardized processing is required before dimensionality reduction processing to facilitate data analysis. In our study, the number of imaging features is large, and not all features are statistically significant, and if not selected for all inclusion, it will cause data redundancy and machine learning model instability. Therefore, it is particularly important to screen out significant radiomics features. In order to evaluate the differences between omics feature delineators in manual delineation, 20 ROIs sketched by two doctors were randomly selected and the intraclass correlation coefficient (ICC) of each feature was calculated. The repeatability of each feature is explained based on the following scores: (1) ICC < 0.4, poor; (2) 0.59 > ICC ≥ 0.40, fair; (3) 0.75 > ICC ≥ 0.6, good; (4) ICC ≥ 0.75, excellent. The characteristics of ICC ≥ 0.75 were considered stable and included in further analytical studies. Then, LASSO regression is used to reduce the dimensionality of radiomics features, which can not only reduce the dimensionality of the data, but also maintain the stability of the established model, calculate the optimal λ value after multiple calculations, and finally screen out the optimal subset for the construction of machine learning models.

### Model construction and evaluation

Using the optimal radiomics characteristics screened by LASSO after dimensionality reduction, six machine learning models were constructed for brain metastasis prediction of lung adenocarcinoma in CT group with CT radiomics combined with EGFR, SUVmax, SUVpeak, Volume, TLG, and PET/CT group with EGFR and PET parameters. They are Logistic Regression (LR), Random forest (RF), Naïve Bayes (NB), Support Vector Machine (SVM), Adaptive Boosting (AdaBoost) and Neural Network**(NN)**. LR is to regress the probability of analyzing the outcome by deriving the loss function from the maximum likelihood and using a logistic function to explain the relationship between a dependent variable and one or more independent variables. RF consists of a large number of individual decision trees that operate as an ensemble, each of which outputs a class prediction, with the most voted categories representing the model’s predictions. NB applies Bayes’ theorem, which assumes conditional independence between features. SVM searches for the optimal spatial separation hyperplane to maximize the boundary. AdaBoost combines multiple weak classifications through a series of learning algorithms, and the output of the learning algorithm is combined to represent the weighted sum of the final output of the enhanced classifier. An NN is a network of highly interconnected processing units that process information through dynamic responses to external inputs. The model was cross-validated with ten folds. The Area under the curve (AUC) is used to evaluate the classification performance of different classifiers. At the same time, the accuracy, sensitivity, and specificity are calculated according to the confusion matrix of the classification results.

### Statistical analysis

Classification of machine learning using Python 3.11.1 software. SPSS 22.0 software performs statistical analysis of data. The chi-square test was used for the analysis of univariate categorical variables, the t-test for the normal distribution of continuous variables was expressed as mean ± standard deviation, and the data that did not conform to the normal distribution were used by the Mann-Whitney U test, and the results were expressed as the median (interquartile range). Multivariate analysis uses the logistic regression model, and the clinically independent predictors in univariate analysis are included in the regression model, where *P* < 0.05 represents a statistically significant difference.

## Results

### Univariate analysis of general data

Univariate analysis showed that there were significant differences in N stage EGFR mutation status and LYM% between the two groups of patients with lung adenocarcinoma (*P* < 0.05). The rate of brain metastases was higher in patients with N2-3 (*P* = 0.018), positive EGFR mutation (*P* < 0.001), and LYM%≤20 (*P* = 0.006) than in the non-brain metastasis group. However, there were no significant differences between the two groups, such as gender, age, primary tumor location, T stage, metastasis at other sites (except brain), white blood cell count, lymphocyte count, neutrophil count, and lymphocyte/neutrophil (*P* > 0.05). See Table 1 for details. Quantitative data analysis showed that CEA(*P* = 0.047), NSE (*P* < 0.001), and Cytra21-1 (*P* < 0.001) were statistically significant in the brain metastasis group and the anencephaly metastasis group. (Table 2)

**Table 1:** Results of univariate analysis of factors affecting general information about cerebral metastasis

Factors		brain metastases		χ2	*P* value
		Not n(%)	Yes n(%)		
gender	Male Female	71(55.5) 57(44.5)	46(60.5) 30(39.5)	0.499	0.480
Age	≤ 60 years >60 years	65(50.8) 63(49.2)	49(64.5) 27(35.5)	3.626	0.057
Location of the primary lesion	Left lung Right lung	51(39.8) 77(60.2)	34(44.7) 42(55.3)	0.470	0.493
T stage	T1-2 T3-4	84(65.6) 44(34.4)	44(57.9) 32(42.1)	1.219	0.270
N stage	N0-1 N2-3	35(27.3) 93(72.7)	10(13.2) 66(86.8)	5.582	0.018
Other metastases(beside brain)	Yes No	39(30.5) 89(69.5)	15(19.7) 61(80.3)	2.822	0.093
EGFR mutation	Yes No	54(42.2) 74(57.8)	53(69.7) 23(30.3)	14.512	<0.001
WBC	≤ 10 × 109/L >10 × 109/L	11(86.7) 17(13.3)	67(88.2) 9(11.8)	0.089	0.766
LYM	≤ 1.1 × 1012/L >1.1 × 1012/L	21(16.4) 10(83.6)	19(25.0) 57(75.0)	2.234	0.135
LYM%	≤ 20% >20%	42(32.8) 86(67.2)	36(47.4) 40(52.6)	4.278	0.039
NEU	≤ 6.3 × 109/L >6.3 × 109/L	10(82.8) 22(17.2)	61(80.3) 15(19.7)	0.209	0.648
NRL	≤ 2.13 >2.13	36(28.1) 92(71.9)	27(35.5) 49(64.5)	1.224	0.269

Note: Note: Categorical variables were expressed as n (%), and intergroup comparisons were made using the χ2 test. The differences in N stage, EGFR mutation status and LYM% between the two groups were significant, with a statistically significant *P* < 0.05

**Table 2:** Results of a univariate Analysis of Tumor Markers Affecting Brain Metastases

Factors	CEA (ng/ml)	NSE (ng/ml)	Cytra21-1 (ng/ml)
No brain metastases	8.93(40.53)	14.75(6.38)	3.785(4.000)
have brain metastases	15.99(80.88)	18.79(12.34)	6.77(7.265)
*Z* value	1.989	4.208	3.590
*P* value	0.047	<0.001	<0.001

### Univariate analysis of PET quantitative indexes

Univariate analysis of PET quantitative indexes showed that SUVmax, SUVpeak, Volume, and TLG were risk factors for brain metastasis of lung adenocarcinoma (*P* < 0.05). The details are shown in Table 3.

**Table 3:** Results of the analysis of quantitative indicators affecting PET for brain metastases

Factors	SUVmean	SUVmax	SUVpeak	Volume	TLG
None brain metastases	3.43(1.64)	10.50(7.18)	8.68(5.44)	37.06(81.14)	129.31(333.92)
Have brain metastases	4.26(0.93)	12.14(7.22)	10.31(0.58)	69.92(117.49)	309.21(532.50)
Z value	-1.953	-2.674	-2.382	-2.345	-2.245
*P* value	0.051	0.007	0.017	0.019	0.025

#### Multivariate analysis of general data

**Fig. 2:**
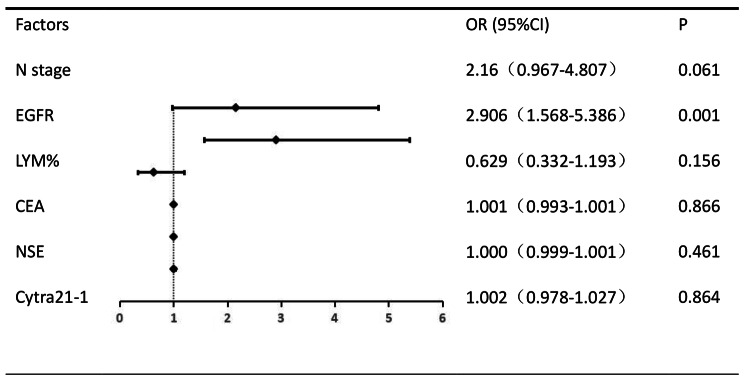
Results of multivariate analysis of general information affecting brain metastases

Multifactorial logistic regression analysis showed that EGFR mutation status was an independent risk factor for brain metastasis in lung adenocarcinoma patients (*P* = 0.001), and the difference was statistically significant. The results of the multifactorial analysis affecting brain metastasis are shown in Fig. 2.

### Results of predictive classification of brain metastasis of lung adenocarcinoma by PET/CTOMICS combined with EGFR based on machine learning

#### Imaging histology feature values obtained by CT after dimensionality reduction

**Fig. 3:**
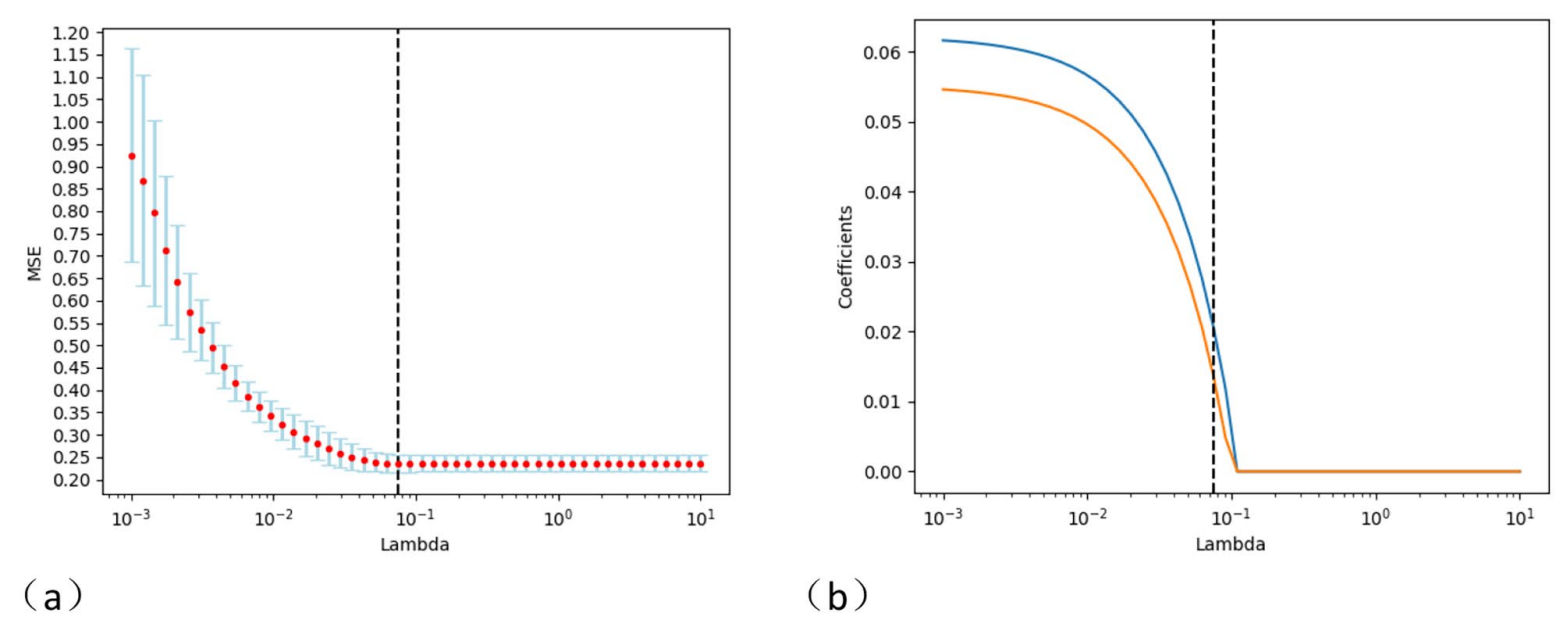
The LASSO method was used to screen CT radiomics features. Note: Fig **a**: MSE path, Represents the mean squared error of different lambda values for each cross-validation;Fig **b**: LASSO path, It indicates the change of the regression coefficient of the independent variable when λ selects different values. Choose optimal λ = 0.07543

Two radiomics features were screened using ICC and LASSO methods (Fig. 3), namely Original GLRLML-Long Run Low Gray Level Emphasis, and Wavelet-LLH GLCM-ClusterTendency. The T-test showed no significant statistical significance, as shown in Table 4 below.

**Table 4:** Lung adenocarcinoma CT radiomics characteristic values

CT radiomics features			None brain metastases	Have brain metastases	T value	*P* value
Original	GLRLM	LongRunLowGrayLevelEmphasis	0.047 ± 0.043	0.046 ± 0.036	0.141	0.888
Wavelet-LLH	GLCM	ClusterTendency	149.87 ± 98.697	142.96 ± 85.08	0.508	0.612

Note: Original: Original features; GLRLM: Grayscale travel matrix; Long Run Low Gray Level Emphasis: Long stroke low grayscale emphasis; Wavelet-LLH: wavelet features; GLCM: Grayscale symbiotic matrix; ClusterTendency: Clustering tendency

#### Radiomics feature values obtained by PET after dimensionality reduction

**Fig. 4:**
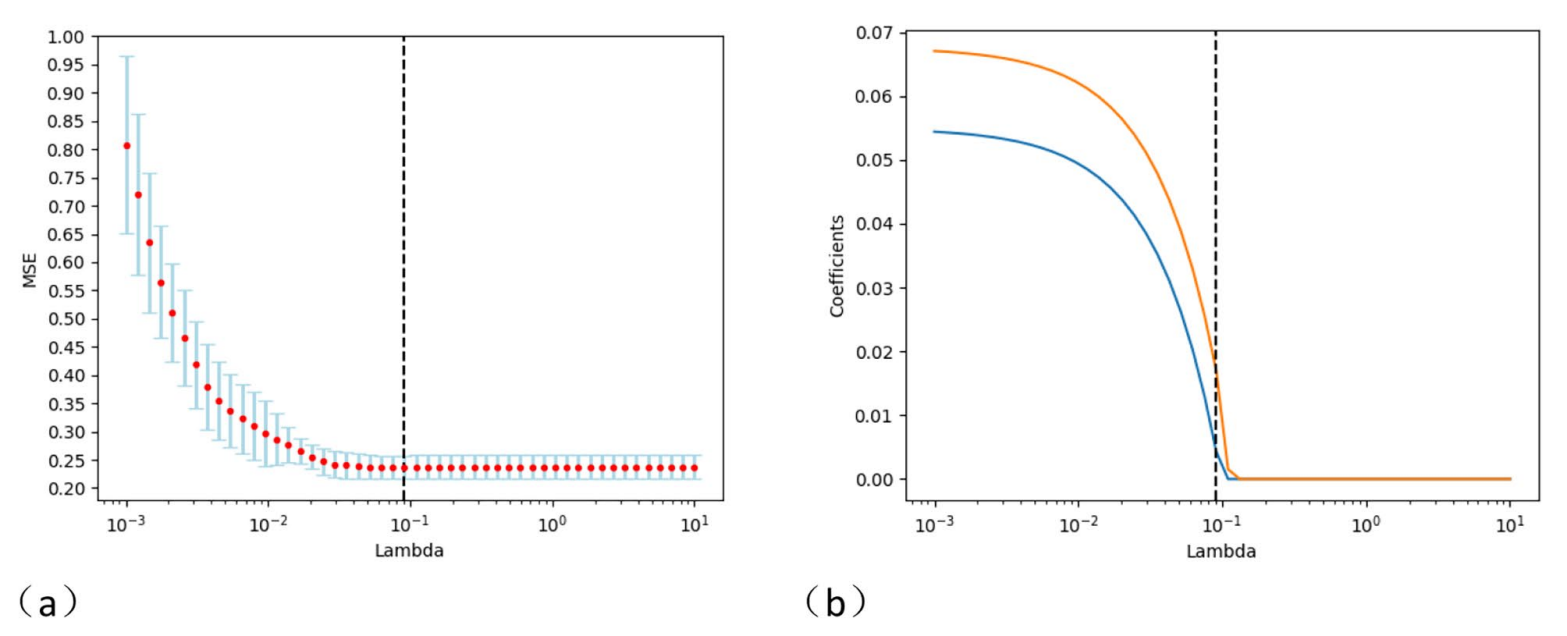
The LASSO method screened the omics features of PET images. Fig **a**: MSE path, Represents the mean squared error of different lambda values for each cross-validation;Fig **b**: LASSO path, Indicates the change in the regression coefficient of the independent variable when λ selects different values. Choose the optimal λ = 0.09103

PET radiomics using ICC and LASSO methods after dimensionality reduction screening also obtained two radiomics features (Fig. 4), Wavelet-HHL GLCM IDN and Wavelet-LLL GLCM Joint Entropy. The t-test showed that all were statistically significant. The 95% confidence intervals are (-0.0191, -0.0048) and (-1.246, -0.325), respectively, as shown in Table 5 below.

**Table 5:** PET radiomics characteristic values of lung adenocarcinoma

PET radiomics features			None brain metastases	Have brain metastases	T value	*P* value
Wavelet-HHL	GLCM	Idn	0.900 ± 0.028	0.912 ± 0.209	-3.283	0.001
Wavelet-LLL	GLCM	JointEntropy	8.475 ± 1.679	9.260 ± 1.493	-3.365	0.001

Note: Wavelet-HHL, Wavelet-LLL: wavelet features; GLCM: grayscale symbiotic matrix; Idn: Normalization deficit; JointEntropy: Joint entropy

### Machine learning model results

**Fig. 5:**
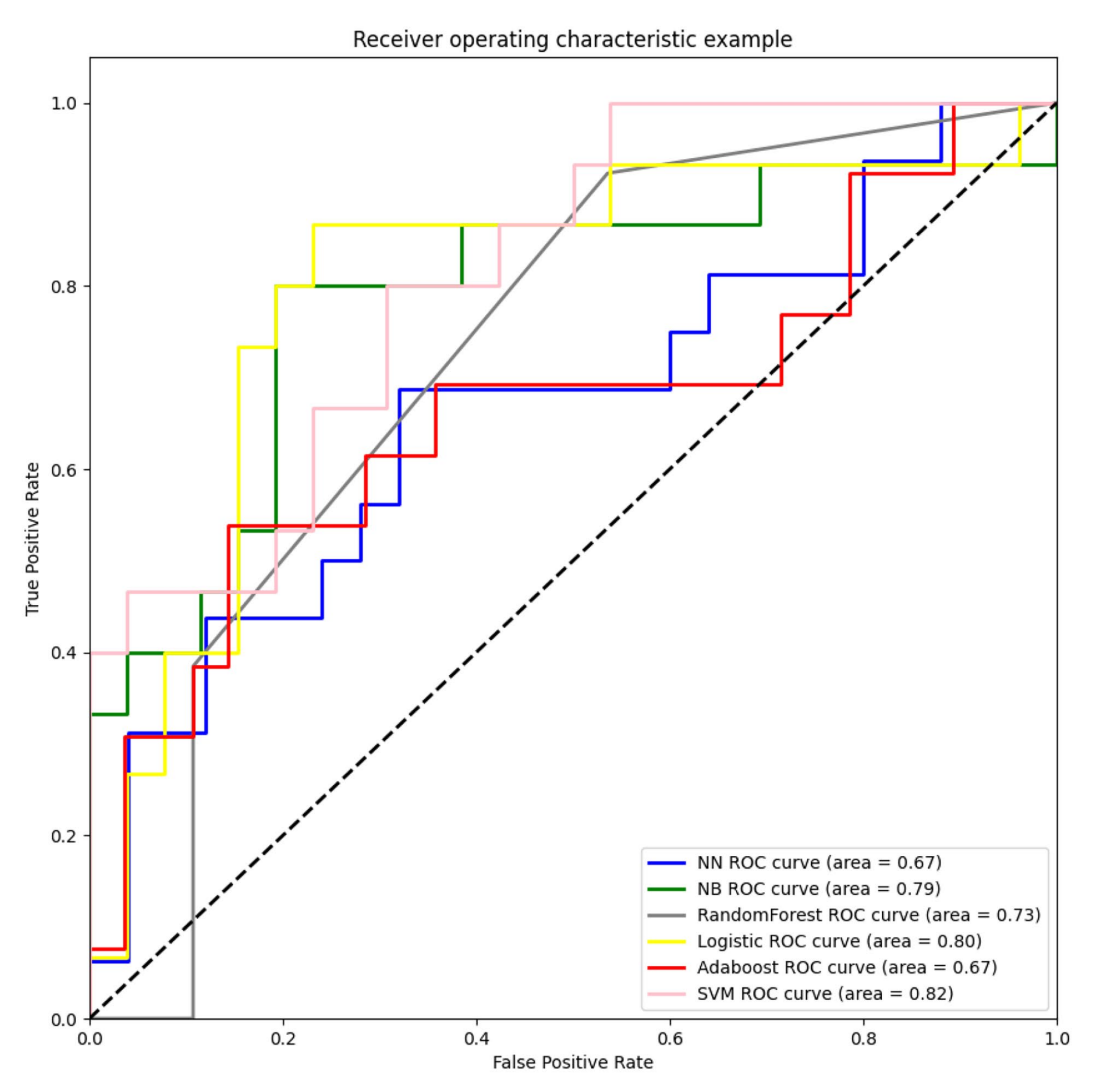
Different models based on PET/CT group predicted ROC curves of brain metastasis of lung adenocarcinoma brain. Note: Receiver operating characteristics (ROC) curves of brain metastasis of lung adenocarcinoma predicted by PET/CT groups of different models. The support vector machine model (SVM) model performed best (AUC = 0.82), followed by the logistic regression model (LR) (AUC = 0.80), the naïve Bayes model (NB) (AUC = 0.79), the random forest model (RF) (AUC = 0.73), the neural network model (NN) (AUC = 0.67), and the adaptive boost model (AdaBoost) (AUC = 0.67). The sensitivity and specificity of the best-performing SVM models were 0.77 and 0.83, respectively

Six machine learning models were constructed for the prediction of lung adenocarcinoma brain metastasis, and the model evaluation parameters were as follows: accuracy, sensitivity, specificity, ROC curve and area under the curve (AUC). The accuracy rates of NN, NB, RF, LR, AdaBoost, SVM models in the group (CT group) based on CT omics characteristics combined with EGFR were 0.61, 0.61, 0.61, 0.68, 0.70, 0.63, sensitivity was 0.50, 0.38, 0.64, 0.67, 0.78, 0.62, specificity was 0.72, 0.68, 0.60, 0.65, 0.64, respectively. The values of 0.78 and AUC were 0.70, 0.68, 0.60, 0.65, 0.64 and 0.70, respectively. Among them, the best predictive brain metastasis model of lung adenocarcinoma was SVM model, with sensitivity and specificity of 62% and 78%, respectively. In the group based on PET radiomics combined with PET quantitative indicators SUVmax, SUVpeak, Volume, TLG and EGFR gene states (PET group), the accuracy rates of NN, NB, RF, LR, AdaBoost, and SVM models were 0.70, 0.61, 0.52, 0.61, 0.61, 0.65, 0.71, and the sensitivity was 0.31, 0.38, 0.54, 0.76, 0.79, respectively. 0.72, the specificity was 0.72, 0.69, 0.50, 0.56, 0.63, 0.80, and the AUC values were 0.61, 0.65, 0.57, 0.70, 0.60, and 0.76, respectively. Among them, the best prediction model for lung adenocarcinoma brain metastasis was SVM model, with sensitivity and specificity of 72% and 80%, respectively. The accuracy rates of NN, NB, RF, LR, AdaBoost, and SVM models in the group (PET/CT group) based on PET/CT radiomics combined with EGFR and PET quantitative indicators SUVmax, SUVpeak, Volume and TLG were 0.63, 0.63, 0.73, 0.66, 0.70, 0.73, and the sensitivity was 0.68, 0.77, 0.89, 0.43, 0.54, respectively. 0.67, the specificity was 0.56, 0.43, 0.38, 0.77, 0.78 and 0.83, and the AUC values were 0.67, 0.79, 0.73, 0.80, 0.67 and 0.82, respectively. Among them, SVM model has the best performance in predicting brain metastasis of lung adenocarcinoma. The results showed that the SVM model performance was more stable and obtained the best indexes in CT group, PET group and PET/CT group, and the prediction performance of PET/CT group was better than that of PET group alone and CT group alone. The sensitivity and specificity of SVM models in the PET/CT group were 77% and 83%, respectively. See Table 6 for details. Among them, the ROC curve of PET/CT group for the evaluation of the efficacy of predicting brain metastasis of lung adenocarcinoma is shown in Fig. 5.

**Table 6:** Machine learning-based radiomics combined analysis of brain metastasis prediction results of lung adenocarcinoma

Model		Accuracy	Sensitivity	Specificity	AUC
Groupe	Machine learning classification				
CT					
	NN	0.61	0.50	0.72	0.70
	NB	0.61	0.38	0.68	0.68
	RF	0.61	0.64	0.60	0.60
	LR	0.68	0.67	0.65	0.65
	AdaBoost	0.70	0.78	0.64	0.64
	SVM	0.63	0.62	0.78	0.70
PET					
	NN	0.70	0.31	0.72	0.61
	NB	0.61	0.38	0.69	0.65
	RF	0.52	0.54	0.50	0.57
	LR	0.61	0.76	0.56	0.70
	AdaBoost	0.65	0.79	0.63	0.60
	SVM	0.71	0.72	0.80	0.76
PET/CT					
	NN	0.63	0.68	0.56	0.67
	NB	0.63	0.77	0.43	0.79
	RF	0.73	0.89	0.38	0.73
	LR	0.66	0.43	0.77	0.80
	AdaBoost	0.70	0.54	0.78	0.67
	SVM	0.73	0.77	0.83	0.82

## Discussion

Although the multidisciplinary treatment of lung adenocarcinoma has significantly improved its overall survival in recent years, the incidence and mortality rate of brain metastases are still high (accounting for 25%∼38% of brain metastases) [20]. Due to the invasive growth of lung adenocarcinoma, brain metastasis is easy to occur hematogenous metastasis, and the non-specific symptoms of brain metastasis lead to late detection of brain metastasis, which is not conducive to the early treatment of patients, makes the disease difficult to control, shortens the survival time of patients and affects their quality of life. Therefore, screening patients with high-risk lung adenocarcinoma who are prone to brain metastasis and early treatment intervention can improve the prognosis of patients. Based on this, this study divided patients with lung adenocarcinoma into brain metastasis group and anencephaly metastasis group, and screened out indicators that may be related to lung adenocarcinoma brain metastasis, such as gender, age, tumor TN stage, serum tumor markers, EGFR mutation status, etc., and included indicators with few previous studies or still controversial indicators, such as lymphocyte percentage, neutrophil-to-lymphocyte ratio, lactate dehydrogenase, etc., to explore the risk factors for lung adenocarcinoma brain metastasis. To explore the value of PET/CT radiomics based on six machine learning models in predicting brain metastasis of lung adenocarcinoma, and to seek the best prediction model, so as to provide early diagnosis basis for brain metastasis in lung adenocarcinoma patients and improve the quality of life and prognosis.

Several studies have reported that both T and N stages in patients with lung adenocarcinoma are predictors of brain metastases, and the incidence of brain metastases increases with the increase of T and N stages [13, 21]. In this study, univariate analysis showed that N stage in lung adenocarcinoma patients was an independent risk factor for brain metastasis, while N stage was not found to be associated with brain metastasis in T stage and multivariate analysis. This may be related to the fact that our study subjects were patients with stage III.-IV. lung adenocarcinoma, excluding patients with early lung adenocarcinoma and no FDG metabolism, so there was a certain difference in the results.

Targeted therapy based on molecular biomarker mutation status has been widely used in the treatment of locally advanced lung adenocarcinoma. In particular, EGFR-positive mutations play an important role in brain metastasis in lung adenocarcinoma [22]. In this study, the rate of brain metastasis in EGFR mutation-positive patients was 69.7%, which was an independent risk factor for brain metastasis of lung adenocarcinoma, and EGFR-positive patients with adenocarcinoma were 2.905 times more likely to develop brain metastasis than EGFR-negative patients. This is consistent with Li’s research [23] that patients with lung cancer with EGFR mutations are more likely to develop brain metastases than patients with EGFR wild-type (OR = 1.99, 95% CI: 1.59–2.48, *P* = 0.000). A possible mechanism [24] involves EGFR activation of epithelial-mesenchymal transformation via protein kinases and STAT3 activation by leukocyte hormone-6 to promote brain metastasis in lung adenocarcinoma. At the same time, it was found in subgroup analysis that the incidence of brain metastasis in patients with L858R site mutation (36.3%) was higher than that in patients with EGFR19 exon mutation (28.3%), and the difference was statistically significant (χ2 = 4.361, *P* = 0.037), and exon 21 point mutation was an independent risk factor for brain metastasis (*P* < 0.05) [21]. Due to the small number of research subjects in this study, and the lack of data on EGFR mutations, the specific mutation types of many cases are not clear, so whether there are differences in different EGFR mutation types in lung adenocarcinoma brain metastasis needs further research and demonstration.

The combined application of multiple tumor markers can improve the sensitivity of auxiliary diagnosis, evaluation of efficacy and prognosis judgment. There is controversy about whether serum tumor markers (CEA, NSE, Cytra21-1) are associated with brain metastases of lung adenocarcinoma. Because elevated serum CEA can play an important role in tumor proliferation and metastasis by inhibiting cell differentiation and promoting tumor angiogenesis [25]. At the same time, CEA-positive tumor cells are more likely to cross the blood-brain barrier and adhere to cerebral blood vessels, thereby promoting the occurrence of brain metastases [26]. In Arrieta et al. [27], there was a significant correlation between high CEA levels and the development of brain metastases, with serum CEA = 40 ng/mL as the boundary (RR = 11.4, 95% CI: 1.7–74, *P* < 0.01).This study also found that serum CEA levels were significantly higher in the lung adenocarcinoma brain metastasis group than in the non-brain metastasis group. At the same time, a [27] study based on patients with locally advanced NSCLC showed that NSE is an independent risk factor for brain metastases. The relationship between elevated NSE levels and brain metastases may reflect tumor heterogeneity or be mediated by neuronal tissue damage around brain metastases, and the specific theoretical mechanism is not clear. Cytra21-1 expression levels are significantly higher in patients with NSCLC brain metastases than in patients without brain metastases and are an independent risk factor for NSCLC brain metastases [28]. Elevated levels of Cytra21-1 have also been found to reduce overall survival, particularly in patients with lung adenocarcinoma [29]. Although we failed to find that serum tumor markers were independent risk factors for lung adenocarcinoma and were different from those mentioned above, there was still a difference between the two groups in univariate analysis (*P* < 0.05), which is similar to previous results,so it can still be speculated that the level of tumor markers can be used to predict the occurrence of brain metastases to some extent. In the later stage, the scope of research objects needs to be expanded for further demonstration.

Regarding the relationship between systemic inflammatory response and tumor prognosis, studies [18, 30] have found that in the early stage of tumor, the aggregation and infiltration of a large number of inflammatory cells provide a superior microenvironment for tumors, promote tumor vascular generation, cell proliferation and metastasis. The comparative study found that the difference between LYN% in the brain metastasis group and the non-encephaly metastasis group was statistically significant, and univariate analysis showed that LYN%≤20 was a risk factor for brain metastasis of lung adenocarcinoma carcinoma, suggesting that low lymphocyte ratio (LYM%≤20) increased the risk of brain metastasis in lung cancer. The relatively stable validation marker NRL was not found to be associated with brain metastases of lung adenocarcinoma in our study. This may be related to the patient’s functional status at admission and whether anti-inflammatory therapy is being administered outside the hospital. At present, few previous studies have reported in this regard, and multi-center and large samples are needed to confirm the relationship between systemic inflammatory markers and brain metastasis of lung adenocarcinoma.

PET/CT can provide anatomical localization of lesions and tumor tissue metabolism information at the same time, which greatly improves the early diagnosis and efficacy evaluation of lung cancer, and is an important imaging technique for lung cancer diagnosis and staging [19, 31, 32]. Some studies have reported [31, 33] that the maximum standard uptake value (SUVmax) in PET is a semi-quantitative indicator of tumor metabolic activity and is an important marker in NSCLC patients. In recent years, the total glycolytic volume (TLG) of lesions has been recognized by more and more people, which represents a comprehensive parameter of tumor metabolic activity and volume, which is helpful to understand the glucose load of lesions. However, it has not been reported whether PET metabolic parameters can predict brain metastases in lung adenocarcinoma patients. Only in previous studies has SUVmax and TLG been reported as semi-quantitative indicators of glucose metabolism with prognostic value for NSCLC, that is, high-level SUVmax has a poor prognosis [34]. In this study, SUVmax, SUVpeak, Volume, and TLG were found to be independent predictors of brain metastasis of lung adenocarcinoma (*P* < 0.05).

Radiomics can reveal tumor microscopic information that cannot be identified by conventional naked eye imaging images, can provide more detailed tumor biology information and tumor microenvironment, and is closely related to gene expression. At present, most of the research on radiomics in the lungs lies in the identification of benign and malignant nodules, gene mutations and molecular phenotypes, tumor case classification and prediction of lung cancer prognosis, and there are few studies in predicting brain metastases. Cong [35] constructed CT omics features and R-scores using eight wavelet-based radiomics features, which were significantly correlated with brain metastases. The optimal AUC of the line graph constructed by combining the R-score and the location of the primary tumor was 0.873 (95% CI: 0.866-0.80) in the validation set, with an average accuracy of 0.827 (95% CI: 0.820–0.834). The correction curve shows that the nomogram prediction results are highly consistent with the actual hidden BM probability (*P* = 0.427). All of the above show that the prediction of brain metastasis by the comprehensive model is better than the results of the omics model. Similarly, based on 204 patients with lung adenocarcinoma, the NN, NB, RF, LR, AdaBoost, and SVM algorithms were used to construct comprehensive models of CT group, PET group, and PET/CT group to explore the value of lung adenocarcinoma in predicting brain metastasis to seek the best prediction model. In the study, it was found that after screening and dimensionality reduction, the characteristic labels of gray symbiosis matrix and grayscale run matrix were retained and related to brain metastasis of lung adenocarcinoma were retained. After dimensionality reduction, the grayscale symbiosis matrix was preserved. This result is consistent with a [36] study of 124 patients with NSCLC who were resected in stages IIB-IIIB with a grayscale symbiosis matrix that plays an important role in brain metastases of NSCLC, with training set AUC = 0.841 (95% CI: 0.754–0.906; *P* < 0.0001), validation set AUC = 0.713 (95% CI: 0.493–0.877; *P* < 0.001)。 That is, the grayscale symbiosis matrix may be of great value in the prediction of brain metastasis in lung adenocarcinoma.

In our study, we explore the application value of six machine learning models in predicting brain metastasis of lung adenocarcinoma and seek their best prediction models. A total of 851 radiomics features of 7 types were extracted from manual and semi-automatic layer-by-layer delineation of the region of interest on chest CT and PET images, mainly including shape features, first-order features, GLDM, GLCM, GLRLM, GLSZM, NGTDM, based on raw data and wavelets. The data were screened and reduced by ICC and LASSO methods, and the two best features were obtained by CT and PET, respectively, and different machine learning models were constructed by CT group using the best imaging omics features of CT combined with EGFR to predict brain metastasis of lung adenocarcinoma. The PET group used the best radiomics characteristics of PET and EGFR, SUVmax, SUVpeak, Volume, and TLG to construct different machine learning models to predict brain metastasis of lung adenocarcinoma. In the PET/CT group, different machine learning models were constructed using CT group and PET group to predict brain metastasis of lung adenocarcinoma. In this study, the first index for evaluating the differential diagnosis performance of the machine learning model was AUC value, and the second index was accuracy. In the CT group, the models with the highest AUC values were SVM model and NN model, but the accuracy of SVM model was higher than that of NN model, so the best model for predicting brain metastasis of lung adenocarcinoma in CT group was SVM model. The SVM model in the PET group had the highest AUC value and was significantly higher than that of other models, so the SVM model in the PET group had the best predictive effect on lung adenocarcinoma brain metastasis. The accuracy of SVM model and RF model in PET/CT group was the same, but the AUC value of SVM model was significantly higher than that of RF model, so the best model for predicting brain metastasis of lung adenocarcinoma in PET/CT group was SVM model.

At the same time, the AUC value of SVM model in PET/CT group was 0.82, which was higher than that of CT group (AUC = 0.70) and PET group (AUC = 0.76). Overall, the performance of the six models in the PET/CT group was better than that of the CT group and the PET group, and the SVM model had the best diagnostic effect, achieving higher sensitivity and specificity (0.77, 0.83). Therefore, PET/CT and EGFR gene testing are necessary in the predictive analysis of brain metastasis of lung adenocarcinoma. Because this is a single-center retrospective study, the sample size is relatively small and external validation of the model is not possible [37]. So we chose tenfold cross-validation instead of dividing cases into training and validation sets. In tenfold cross-validation, we divide the data into 10 equal-sized subsets and perform 10 network trainings, each missing a subset from the training. The average of the accuracy of the 10 results is used as an estimate of the accuracy of the algorithm. Therefore, we do not yet have a clear model that can be further tested in external groups. However, our study preliminarily demonstrates the feasibility of machine learning-based PET/CT radiomics combined with EGFR analysis in predicting brain metastasis of lung adenocarcinoma. Multicenter studies with larger sample sizes and external validation are needed to build models and obtain stronger evidence before clinical application [38]. In the follow-up research process, we will continue to explore the application value of machine learning in the prediction of lung adenocarcinoma brain metastasis, and obtain better models.

## Conclusions

Positive EGFR mutation is an independent risk factor for brain metastasis in patients with lung adenocarcinoma and has important clinical guiding significance. Radiomics combined with EGFR machine learning model is a new method to predict brain metastasis of lung adenocarcinoma. The predictive value of different machine learning models for brain metastasis of lung adenocarcinoma was discussed, among which the best models for predicting brain metastasis of lung adenocarcinoma in CT group, PET group and PET/CT group were all SVM models, and the prediction performance of PET/CT group was better than that of PET alone group and CT alone group.

## Data Availability

Data is provided within the manuscript or supplementary information files.

## Abbreviations

MST: Median survival time
ROI: Region of interest
PCI: Prophylactic cranial irradiation
DFS: Disease free survival
EGFR: Epidermal growth factor receptor
WBC: White blood cell
NEU: Neutrophil
LYM: Lymphocyte
NRL: Neutrophil lymphocyte ratio
CEA: Carcinoembryonic antigen
NSE: Neuron-specific enolase
ICC: Intragroup correlation coefficient
GLDM: Gray level dependence matrix
GLCM: Gray level cooccurrence matrix
GLRLM: Gray level run length matrix
GLSZM: Gray level size zone matrix
NGTDM: Neighboring gray tone difference matrix
LASSO: Least absolute shrinkage and selection operator
SUV: Standard uptake value
TLG: Total lesion glycolysis
BM: Brain metastases
NN: Neural network
NB: Naive Bayes
RF: Random forest
LR: Logistic Regression
AdaBoost: Adaptive Boosting
SVM: Support Vector Machine
